# Contribution of the FilmArray BioFire® Technology in the Diagnosis of Viral Respiratory Infections during the COVID-19 Pandemic at Ibn Sina University Hospital Center in Rabat: Epidemiological Study about 503 Cases

**DOI:** 10.1155/2023/2679770

**Published:** 2023-06-20

**Authors:** Khalid Edderdouri, Hakima Kabbaj, Leila Laamara, Noureddine Lahmouddi, Oumayma Lamdarsi, Amal Zouaki, Ghizlane El Amin, Jalila Zirar, Myriam Seffar

**Affiliations:** ^1^Mohamed V University, Faculty of Medicine and Pharmacy, Rabat, Morocco; ^2^Ibn Sina University Hospital Center, Central Laboratory of Virology, Rabat, Morocco

## Abstract

Respiratory viruses are the most involved pathogens in acute respiratory infections. During the COVID-19 pandemic, new elements have been brought to this topic, especially at the diagnostic and therapeutic level. The objective of this work is to describe the epidemiology of respiratory viruses in patients admitted to the Ibn Sina University Hospital of Rabat during a period characterized by the emergence and spread of SARS-CoV-2. We conducted a retrospective study from January 1 to December 31. We included all patients treated for acute respiratory infection and for whom a multiplex respiratory panel PCR was requested. Virus detection was performed using a FilmArray RP 2.1 plus BioFire multiplex respiratory panel. The study population was relatively adults with a mean age of 39 years. The sex ratio M/F was 1.20. The survey revealed a high prevalence of 42.3% of patients hospitalized in the adult intensive care unit whose respiratory distress was the most common reason for hospitalization (58%). The positivity rate was 48.1%. This rate was higher in the pediatric population 83.13% compared to adults 29.7%. Monoinfection was found in 36.4% of cases, and codetection in 11.7% of cases. This survey revealed that a total of 322 viruses were detected, HRV being the most incriminated virus (48.7%), followed by RSV in 13.8% of patients. Considering the five most detected viruses in our study (HRV, RSV, PIV3, ADV, and hMPV), we found that the incidence was significantly higher in the pediatric population. SARS-CoV-2 was detected only in adult's population. In our study, we found that influenza A and B viruses, PIV2, MERS, and all bacteria were not detected by this kit during the study period. Regarding the seasonal distribution, RSV and hMPV showed a significantly high incidence during autumn and summer and SARS-CoV-2 and CoV OC43 showed a high peak during winter. In this study, we found a lack of detection of influenza virus and a shift in the usual winter peak of RSV to the summer, while the detection of ADV and HRV was less affected. This difference in detection could be due on the one hand to the difference in stability between enveloped and nonenveloped viruses and on the other hand to the escape of certain viruses to the different sanitary measures introduced after the declaration of the COVID-19 pandemic. These same measures were effective against enveloped viruses such as RSV and influenza viruses. The emergence of SARS-CoV-2 has modified the epidemiology of other respiratory viruses, either directly by viral interference or indirectly by the preventive measures taken.

## 1. Introduction

Acute respiratory infections (ARI) represent a public health problem. They are responsible for significant mortality all over the world, especially in children aged less than 5 years [1]. ARI represent a challenge for health systems, especially in developing countries.

Respiratory viruses are the predominant cause of ARI [2]. Their role is of growing interest, especially with the evolution of molecular methods and more particularly multiplex methods that allow the detection of many infectious agents simultaneously, with high sensitivity and specificity. These molecular diagnostic methods based on the syndromic approach in the detection of respiratory pathogens are increasingly used and it allows a rapid distinction between viral and bacterial infections, in particular by the FilmArray® BioFire multiplex Respiratory Panel (FA-RP). This panel allows detection of respiratory viruses, including SARS-CoV-2 and some bacteria, in less than one hour. This method is sensitive (>80%), reproducible with better detection of coinfections [3]. The principle of the reaction is based on nested PCR with melting curve analysis.

Coronavirus disease 2019 (COVID-19) is a global pandemic that first appeared and was reported in Wuhan city, China, in December 2019. It is caused by severe acute respiratory syndrome coronavirus 2 (SARS-CoV-2). The virus invades the targeting organs such as the alveolar epithelial cells by binding the S1 domain of the viral spike protein to cellular receptor angiotensin-converting enzyme 2 (ACE2). COVID-19 is usually asymptomatic or presents flu-like symptoms. But it may complicate with a more serious course. Several observational studies have shown that aberrance of immune-inflammatory response and development of cytokine storm might be the reasons behind multiorgan and dead ends of COVID-19 [4, 5].

The COVID-19 pandemic has brought new elements to the subject of ARI, especially at the level of diagnostic and therapeutic, representing a real challenge for the Global Health System. Indeed, following the emergence and widespread of SARS-CoV-2 since 2019, preventive measures have been taken almost everywhere in the world, such as the wearing of masks, hand hygiene, physical distancing, confinement, and others. These measures have affected the epidemiology of other respiratory viruses which have the same mode of transmission. It is therefore important to assess the incidence of respiratory viruses in search of a possible change following the introduction of SARS-CoV-2 in the context of a future cocirculation of this virus with other respiratory viruses.

The aim of this retrospective study is to describe the epidemiology of different respiratory viruses in patients admitted to Ibn Sina University Hospital of Rabat during a period characterized by the emergence and widespread of SARS-CoV-2 and to highlight the role of multiplex real-time PCR in the rapid diagnosis of ARI.

## 2. Materials and Methods

### 2.1. Study Design and Clinical Specimens

A retrospective study was carried out in the Central Virology Laboratory (CVL) of Ibn Sina University Hospital, Hospital of Specialties, Rabat, including patients treated for acute respiratory tract infection and hospitalized in different services of Ibn Sina University Hospital, and for whom a multiplex PCR respiratory panel was requested.

Samples which came in CVL from January 1 to December 31, 2021, were taken as the study population.

Patients with clinical symptoms (included fever and/or cough and/or other symptoms suggestive of respiratory infection: rhinorrhea, nasal congestion, or sore throat) were evaluated during this study. The majority of the samples were collected by using a nasopharyngeal swab and transported to the laboratory (CVL) in a transport medium (SOTHEMA® or Pharma5®) containing sterile saline.

Baseline clinical data and the epidemiological characteristics of each patient were collected using a dedicated form. The data collected were age, gender, department, date of hospitalization, date of onset of symptoms, and clinical symptoms. Comorbidities (cardiovascular disease, chronic lung disease, diabetes, pregnancy, chronic respiratory failure, hematological disease, and chronic neurological disease) and the patient's vaccination status against the influenza virus were also collected.

Virus detection was performed using a multiplex respiratory panel, FilmArray RP 2.1 plus BioFire multiplex respiratory panel. This panel allows simultaneous detection of viruses and bacteria in less than one hour [3]. The principle of the reaction is based on nested PCR with melting curve analysis. The cassettes were prepared by injecting 1 ml of the hydration solution and 300 *μ*l of the sample combined with its buffer. Then, the cassette is placed in the FilmArray system and the analysis program is started. It is a unitary, closed, disposable system that contains all the chemical reagents necessary to isolate, amplify, and detect nucleic acids of multiple respiratory viruses and bacteria in a single sample. We notice that multiplex PCR is targeting both viral and bacterial pathogens. The list of pathogens detectable by this panel includes 19 viruses: Adenovirus (ADV), Coronavirus 229E (CoV 229E), Coronavirus HKU1 (CoV HKU1), Coronavirus NL63 (CoV NL63), Coronavirus OC43 (CoV OC43), MERS-CoV, SARS-CoV-2, Metapneumovirus (hMPV), Influenza A Virus (IAV), Influenza A/H1, Influenza A/H1-2009, Influenza A/H3, Influenza B virus (IBV), parainfluenza viruses 1 to 4 (PIV1-4), Human rhinovirus/Enterovirus (HRV), respiratory syncytial virus (RSV), and 4 bacteria (*Bordetella pertussis*, *Bordetella parapertussisMycoplasma pneumoniae, and Chlamydia pneumoniae*).

### 2.2. Data Treatment and Statistical Analysis

Analysis of the results was performed using SPSS version 21 software (SPSS Inc., Chicago, III, USA). The difference between the ratios was evaluated using the chi-square test and Fisher's exact test. *P* < 0.05 was considered statistically significant.

## 3. Results

### 3.1. Characteristics of the Study Population

We retrospectively analyzed the results of 503 samples with age ranging between 1 week newborn to 93 years with an average age of 39 years. The sex ratio M/F was 1.20. Adults represented 325 (64.6%) patients, while 178 (35.4%) patients were children aged less than 15 years. Age distribution of the study population is given in Table 1. We found that 42.3% of cases (*n* = 213) were hospitalized in the adult intensive care unit, while 22.2% (*n* = 112) were hospitalized in other adult medical units, 7.1% (*n* = 36) in the pediatric intensive care unit, and 28.3% (*n* = 142) in the other pediatric medical units.

Nasopharyngeal swabs were the most used (98.6%, *n* = 496). For the rest of the samples, a protected distal swab was used in 0.8% (*n* = 5) and bronchial aspiration in 0.4% (*n* = 2).

Respiratory distress was the most common reason for hospitalization (58%) in this study, followed by lung disease (16%) and asthma (4.5%). Reasons of hospitalization are given in Table 1.

### 3.2. Virological Profile of the Collected Patients

Among the 503 patients treated, 242 patients were tested positive for a total positivity rate of 48.1%. Of the 242 positive cases, 183 (36.4%) samples were positive for viral respiratory single infection and 59 (11.7%) as codetection.

The positivity rate according to age was statistically significant (*P* < 0.001). In our study, we have found a high incidence of children (*n* = 148/178, 83.13%) compared to adults (*n* = 94/325, 29.7%). The positivity rate according to age and mode of infection is given in Table 2.

**Figure 1:**
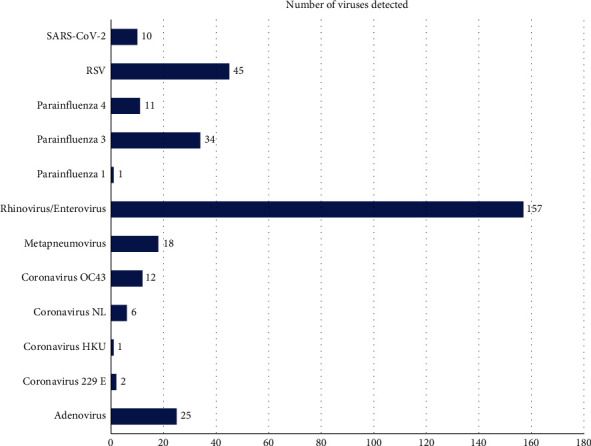
Distribution of the different viruses detected by the FilmArray RP 2.1 kit Plus.

Considering all of the 322 pathogens detected from clinical samples, HRV was the predominant respiratory pathogen isolated in 157 patients (48.7%), followed by RSV which was found in 45 patients (13.8%). The distribution of the different pathogens is given in Figure 1.

Considering the five most detected viruses in our study (HRV, RSV, PIV3, ADV, and hMPV), we found that the incidence was significantly higher in the pediatric population (*P* < 0.0001 for the five viruses). Among the isolated pathogens, ADV was mostly detected in children aged less than 5 years, while SARS-CoV-2 was detected only in adult's population.

In our study, we found that influenza A and B viruses, PIV2, MERS, and all bacteria were not detected by this kit during the study period. Distribution of viruses according to age is given in Table 3.

In our study, we found 59 (11.7%) cases of codetection, which represent 24.4% of positive samples.

Among these 59 cases, we found that 44 (74.5%) samples were positive for two viruses, 12 (20.5%) samples were positive for three viruses, 2 (3.5%) samples were positive for four viruses, and one sample (1.5%) was positive for five viruses.

**Figure 2:**
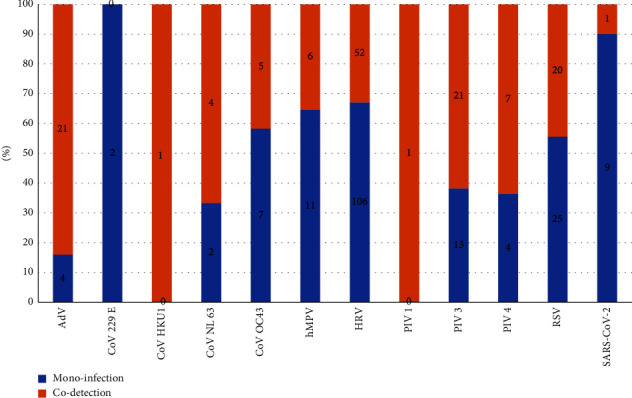
Distribution of viruses according to the mode of infection.

The codetection HRV + ADV was predominant (22%, 10/44), followed by HRV + PIV3 (20%, 9/44) and HRV + RSV (18% 8/44). For all codetections, 81.3% were isolated in children with a significantly higher rate compared to adults (48/178 children or 27%, 11/325 adults or 3.4%, *P* < 0.0001). Among the most involved viruses in codetection, HRV was mostly found (78%, 46/59), followed by PIV3 and ADV (32%, 19/59 for both), RSV (30%, 18/59), and PIV 4 (15%, 9/59) (Figure 2 and Table 4).

Regarding the seasonal distribution of the detected viruses, there was no significant difference in the positivity rate between winter (40.3%), spring (45.7%), summer (46.8%), and autumn (58.8%) (*P*=0.020).

**Figure 3:**
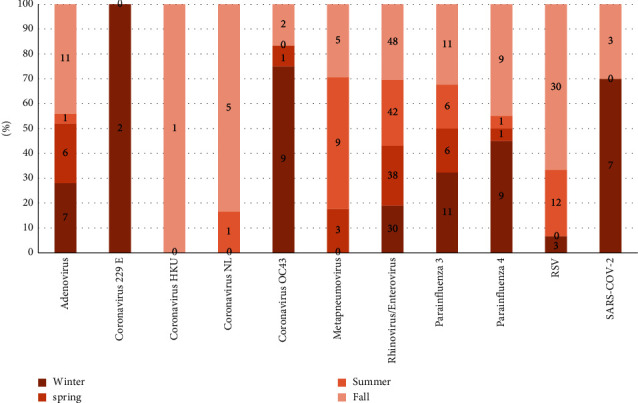
Seasonal distribution of viruses.

HRV and PIV3 were detected all over the year, with no seasonality. RSV and hMPV showed a significant difference during the season with high incidence during autumn and summer (*P* < 0.0001 and *P*=0.005, respectively). PIV 4 showed a winter peak that was statistically significant (*P*=0.001). SARS-CoV-2 and CoV OC39 also showed a similar peak during the cold season (*P*=0.005 and *P*=0.002, respectively) (Figure 3 and Table 5).

## 4. Discussion

Viruses remain the most implicated pathogens in acute respiratory infections (ARI) with high morbidity and mortality, particularly in children. It is therefore imperative to understand the etiology and epidemiology of these viruses in order to control and prevent these ARIs [6]. In this study, we described and analyzed the epidemiological data of respiratory viruses in patients hospitalized at Ibn Sina University Hospital in Rabat over 12 months covering the year 2021. This period was characterized by the emergence and global spread of SARS-CoV-2 since the end of 2019 [7].

In our study, 496 (98%) clinical samples coming from patients admitted in different departments of Ibn Sina University Hospital in Rabat were analyzed by using a nasopharyngeal swab. Indeed, this sampling remains more practical and easier to perform and allows a detection of viruses at a very high rate.

Our total positivity rate was 48.1%, which is higher than the other studies using the same method; Brittain-Long et al. [8], Çiçek et al. [9], Mandelia et al. [10], and Da Silva et al. [11] reported a positivity rate between 30 and 33.4%. Ambrosioni et al. [12] and Huang et al. [3] reported a positivity rate of 43.2% and 44.5%, respectively. Marcil et al. in a study conducted in our laboratory in 2015-2016, reported a higher positivity rate of 65% [13]. The positivity rate was high because the test was only conducted on patients treated for acute respiratory tract infection and hospitalized in different services especially in resuscitation.

Our study confirms that children are a vulnerable population compared to adults with a higher positivity rate of 83.13%. Because of their physical and immune weakness, they are susceptible to rapidly transmitted and highly contagious viruses [14]. Similar positivity rates have been noted in other studies [14, 15]. Although the positivity rate is higher in children, the need for care in intensive care units is higher in adults with more severity and mortality factors.

Our study shows that HRV was the predominant respiratory pathogen detected (48.75%), followed by RSV, PIV3, and ADV, which is in accordance with the literature. Brittain-Long et al. and Sentilhes et al. reported that HRV was the most detected virus (38%, 35%), followed by RSV (13.5% and 26%) and influenza viruses (10.5% and 12%) [8, 16]. Compared to other pathogens, the rate of SARS-CoV-2 was lower. Indeed, our laboratory used another test for the detection of the SARS-CoV-2 genome: MAScIR-CoV-2M Kit 2.0.

HRV was the most detected virus in our study (*n* = 157). It was predominant in both mono- and codetection. While there was a difference in the predominant pathogen in different age categories, HRV was predominant in children (*P* < 0.0001). Earlier studies reported that HRV was the most involved virus in respiratory tract infections, especially in children [17]. Nevertheless, the clinical significance of this detection by highly sensitive multiplex PCR is questioned. This is due to the detection of this virus in asymptomatic subjects [10, 18]. In addition to this statement, rhinovirus can be detected as positive due to prolonged viral shedding especially in children. Regarding the seasonal distribution in our study, HRV was detected all over the year (*P*=0.80). Similar result has been reported in other studies [16, 19, 20].

RSV was the second most detected virus in our study with a total of 45 cases and a peak during autumn. RSV was the predominant pathogen in children; we note a significant statistically difference (*P* < 0.0001) between the two age groups. RSV is considered as the main cause of hospitalization and an important factor that leads to child mortality [21, 22]. Our result is in accordance with other studies [3, 21, 23]. For the seasonal distribution, each virus has a seasonal peak; the winter peak is usually characteristic of RSV virus, which has been confirmed by many studies [16, 17, 24, 25]. However, in our study, RSV showed a peak in autumn with a statistical significance (*P* < 0.0001). RSV showed a seasonal peak that extends later than the usual seasonal activity. This finding can be explained by the fact that the application of preventive measures for the COVID-19 pandemic is likely and may have delayed the epidemic of this virus, with reappearance after relaxing measures.

In this study, 46 patients were tested positive for PIVs (14.28%), of which 34 were positive for PIV3 (73.9%), 11 for PIV4 (23.9%), and 1 for PIV1 (2.2%). The same result has been reported in other studies [26, 27]. PIV3 was mostly detected in children (85.3% of PIV3 positive cases) with statistical significance (*P* < 0.0001). PIV3 did not show a specific seasonality. In contrast, PIV4 showed a winter peak with statistical significance (*P* < 0.0001).

ADV was detected in 25 patients. In addition, 17 patients were positive for hMPV. The prevalence of both virus' infection was statistically higher in children, *n* = 19/25 (76%) for ADV and *n* = 16/17 (94.7%) for hMPV (*P* < 0.0001 for both). ADV was particularly prevalent in children aged less than 5 years. Similar results have been observed in other studies [14–17]. Both viruses did not show a specific seasonality. Indeed, they were detected throughout the year [19, 28].

In addition, 31 coronaviruses were detected. CoV OC43 (*n* = 11) was the most frequently isolated, followed by SARS-CoV-2 (*n* = 10) and CoV NL63 (*n* = 6). The age-dependent positivity rate was not statistically significant for these viruses. Regarding the seasonal distribution of these viruses, SARS-CoV-2 and CoV OC43 are known to have a high incidence of infection during winter with a significant difference (*P*=0.005 and *P*=0.002, respectively) [28, 29].

Coinfection is common in respiratory tract infections [3, 10]. Multiplex PCR can detect and significantly estimate the prevalence of viral coinfections [30]. In our study codetection was observed in 11.7% of cases, with a statistically significant rate in children (19.8% of positive test results) compared to adults (4.5%) (*P* < 0.0001). Similar results were reported by several respective studies provided by Ambrosioni et al. [12], Huang et al. [3], and Mandelia et al. [10]. In our study, the codetection of two viruses was the most common, followed by the codetection of 3 viruses.

Other rare cases showed the codetection of 4 and 5 viruses. HRV was the most involved virus in codetection, which can be explained by its high propagation during the study period. This high rate of codetection can be explained by using a highly sensitive multiplex molecular method that can detect several respiratory pathogens simultaneously. The rate of codetection with SARS-CoV-2 was very low; in fact, the replication of SARS-CoV-2 would be reduced by the presence of other respiratory viruses, especially with HRV, as described in an English study [31]. Furthermore, these codetections may correspond to coinfection, sequential infection, contamination, or cross-reaction. Hence, the fact that in the absence of quantitative results, these results remain insufficient, hence the interest of the threshold cycle (Ct) which provides a semiquantitative estimate of the viral load.

Indeed, the Cts exist on the FilmArray but they are not directly accessible to the operator. They are only available on request to the manufacturer (Biomérieux). The strict respect of the directives concerning the operating mode and the hygiene measures fixed by the supplier is essential in order to avoid any contamination.

Of the 25 cases detected with ADVs in our study, 21 (84%) cases were involved in codetection with a significant difference (*P* < 0.0001). This is in concordance with a study provided by Midgley et al.who found that the rate of codetection of ADV is high [25]. ADV has a prolonged shedding time due to its stable DNA genome, suggesting that the stability of this genome is responsible for the increased frequency of codetection [10]. In addition to being nonenveloped, rhinovirus and adenovirus are morphologically smaller than others according to basic virology knowledge.

Influenza virus was not found in this study, while Marcil et al., in a study conducted in our CVL laboratory, found 21 (17%) cases of influenza infection, including 16 cases of influenza A (H1N1), 2 cases of influenza A non-H1N1, and 3 cases of influenza B [13]. However, this study was carried out before the advent of the COVID-19 pandemic. Moreover, other studies have shown similar results to ours. A study conducted in Brazil during the COVID-19 pandemic has not found any cases of influenza virus [31]. A similar result was found in the United Kingdom and Australia with a very low rate of influenza virus cases during the 2020-2021 winter season [32, 33]. Among the hypotheses that have been proposed to explain the very low incidence of influenza virus during the COVID-19 pandemic are the use of preventive measures for COVID-19 pandemic control, such as masking, school and workplace closures, physical distancing, and others. These measures are likely to have been effective in limiting the spread of influenza viruses and possibly other respiratory pathogens. Influenza virus and SARS-CoV-2 are both primarily transmitted in much the same way via respiratory droplets. The low contagiousness of influenza virus (*R*0 = 1.28) compared to SARS-CoV-2 (*R*0 = 2–2.5) probably had limited transmission of flu [34]. Nevertheless, these results indicate that the reduction of influenza viral transmission can potentially affect the immunity of the population, which could make it more vulnerable in the following season [33]. Moreover, it is described that one respiratory virus can block infection with another through stimulation of antiviral defenses [35]. This is the case of rhinovirus infection, which by inducing an antiviral response to interferon, will allow immunological protection against influenza virus infection, which may reduce the spread of influenza viruses [35]. In the same context, several studies have noted that the rhinovirus could have hindered the spread of the influenza A (H1N1) virus during the influenza A (H1N1) pandemic in 2009 [36]. The worldwide implementation of different preventive measures in order to hinder the spread of SARS-CoV-2 virus has influenced the activity of other respiratory viruses. In this study, we note an absence of influenza virus cases and a change in RSV seasonality, while the detection of ADV and HRV was very little affected. This finding can be explained by the difference in stability between enveloped and nonenveloped viruses [37]. Nonenveloped viruses, such as HRV and ADV, are more resistant to disinfectants containing ethanol (among others) and can survive for extended periods on surfaces [38]. Moreover, it is reported that surgical masks were not able to completely block the transmission of rhinovirus [39], which could explain the escape of these viruses from the preventive measures during COVID-19 pandemic. These same measures were effective against enveloped viruses such as RSV and influenza viruses.

## 5. Conclusion

The COVID-19 pandemic has highlighted the risk of respiratory infections especially in vulnerable patients. The emergence of SARS-CoV-2 has modified the epidemiology of other respiratory viruses, either directly by viral interference or indirectly by preventive measures taken almost everywhere in the world, to hinder this emergence.

Multiplex PCR in general and the FilmArray BioFire® technology, in this case, has contributed to describe the epidemiology of different respiratory viruses during a period characterized by the emergence and widespread of SARS-CoV-2 virus. The improvement of diagnostic tools, in particular molecular biology methods, brings another dimension to its syndromic approach, thus allowing a better understanding of the epidemiology of respiratory viruses.

## Figures and Tables

**Table 1:** Characteristics of the study population.

	Number		Percentage (%)

*Age of patients*			
Adult	325		64.6
Child	178		35.4
Total	503		100.0

*Gender*			
Male	275		54.6
Female	228		45.4
Total	503		100.0

*Service*			
Adult reanimation	216		42.9
Adult medical service	213		22.5
Pediatric resuscitation	33		6.6
Pediatric medical service	141		28.0
Total	503		100.0

*Admission season*			
Winter	134		26.6
Spring	107		21.2
Summer	126		25.1
Autumn	136		27.1
Total	503		100

*Reason for hospitalization*			
Respiratory distress	293		58.2
Pulmonary disease	81		16.1
Asthma	23		4.6
Diabetic ketoacidosis	8		1.6
Heart disorder	6		1.2
Not specified	46		9.15
Total	457		

*Clinical signs and symptoms*			
Symptoms	Presence of the symptoms	Absence of the symptoms	Not specified
Cough	259	231	13
Fever	270	220	13
Rhinorrhea	66	424	13
Breathing difficulty	203	287	13

**Table 2:** Positivity rate according to age and mode of infection.

			Number	Percentage^*∗*^ (%)
*Results*				
Negative	Adult	231	261	51.9
	Child	30		

Positive	Adult	94	242	48.1
	Child	148		

Total			503	100.0

*Mode of infection*				
Monoinfection	Adult (%)	83 (55.4)	183	36.4
	Child (%)	100 (54.6)		

Codetection	Adult (%)	11 (18.7)	59	11.7
	Child (%)	48 (81.3)		

Total			242	48.1

^*∗*^Of the total cases.

**Table 3:** Distribution of viruses according to age.

Virus	Child (%)	Adult (%)	*P* value
RSV	36 (80)	9 (20)	<0.0001
ADV	19 (76)	6 (24)	<0.0001
CoV 229E	0 (0)	2 (100)	0.542
CoV HKU1	0 (0)	1 (100)	1.00
CoV NL63	2 (33.33)	4 (66.64)	1.00
CoV OC43	5 (41.66)	7 (58.34)	0.761
hMPV	17 (94.4)	1 (4.6)	<0.0001
HRV	100 (63.7)	57 (36.3)	<0.0001
PIV1	1 (100)	0 (0)	0.354
PIV3	29 (85.3)	5 (14.7)	<0.0001
PIV4	8 (72.7)	3 (27.3)	0.20
SARS-CoV-2	0 (0)	10 (100)	0.17
Total	217 (71.7)	105 (28.3)	—

**Table 4:** Distribution of codetections.

Co-détection virale	Numbers of case
HRV + PIV4	3
HRV + RSV	8
HRV + PIV3	9
HRV + ADV	10
HRV + CoV OC43	1
HRV + SARS-CoV-2	1
HRV + CoV HKU1	1
HRV + CoV NL63	1
HRV + CoV OC43	1
ADV + RSV	5
ADV + PIV4	2
RSV + CoV NL63	1
HRV + ADV + RSV	2
HRV + ADV + hMPV	3
HRV + CoV OC43 + PIV4	1
HRV + hMPV + PIV3	2
HRV + RSV + PIV3	1
HRV + PIV3 + PIV4	2
PIV1 + PIV3 + RS	1
HRV + hMPV + CoV NL63 + PIV3	1
ADV + CoV OC43 + HRV + PIV3	2
ADV + CoV OC43 + HRV + PIV3 + PIV4	1

**Table 5:** Virus prevalence according to the season.

Virus	Autumn	Winter	Spring	Summer	*P* value
RSV	30 (66.66%)	3 (6.66%)	0	12 (26.68%)	<0.0001
ADV	11 (44%)	7 (28%)	6 (24%)	1 (4%)	0.31
CoV 229E	0	2 (100%)	0	0	0.178
CoV HKU1	1 (100%)	0	0	0	1.00
CoV NL63	5 (83.33%)	0	0	1 (16.67%)	0.16
CoV OC43	2 (16.66%)	9 (75%)	1 (8.34%)	0	0.002
hMPV	10 (55.50%)	0	3 (16.70%)	5 (27.8%)	0.005
HRV/Enterovirus	47 (29.9%)	30 (19.1%)	38 (24.20%)	42 (26.8)	0.80
PIV1	1 (100%)	0	0	0	1.00
PIV3	11 (32.30%)	11 (32.30%)	6 (17.7%)	6 (17.7%)	0.606
PIV4	0	9 (81.8%)	1 (9.1%)	1 (9.1%)	0.001
SARS-CoV-2	3 (30%)	7 (70%)	0	0	0.005

## Data Availability

The data for the current study are available from the corresponding author on reasonable request.
